# “Value Beyond Management”: Professional Transition Among Senior Head Nurses in China: A Descriptive Qualitative Study Based on Schlossberg’s 4S Model

**DOI:** 10.1155/jonm/1907433

**Published:** 2026-10-23

**Authors:** Yucheng Gao, Yao Luo, Jiajia Liu, Gang Gan, Nina Wang, Xiaochen Yan, Xiaoyu Du, Xincen Liu, Wendi Guo, Wenjiao Li, Wenjun Chen, Jia Chen

**Affiliations:** ^1^ Xiangya School of Nursing Central South University Changsha Hunan China csu.edu.cn; ^2^ Xiangya Third Hospital Central South University Changsha Hunan China csu.edu.cn; ^3^ Xiangya Hospital Central South University Changsha Hunan China csu.edu.cn

**Keywords:** adaptation, career mobility, nurse administrators, nursing management, psychological, qualitative research

## Abstract

**Background:**

As the nursing workforce ages, an increasing number of senior head nurses are transitioning from managerial to nonmanagerial roles. However, little is known about how these nurses experience and navigate this professional transition, despite its important implications for workforce sustainability, retention, and knowledge transfer within healthcare organizations. Guided by Schlossberg’s 4S transition model, we aim to explore the transition situations, individual traits and psychological resources, external support, and adaptation strategies of senior head nurses transitioning from managerial to nonmanagerial roles.

**Methods:**

A descriptive qualitative design was employed. Eighteen female senior head nurses who had recently stepped down from managerial positions were recruited from four tertiary public hospitals in central China using purposive and maximum variation sampling. Data were collected through semistructured interviews and analyzed using inductive thematic analysis. Schlossberg’s 4S transition model was subsequently used as a theoretical lens to interpret the inductively derived themes.

**Results:**

Four themes were identified through inductive thematic analysis: (1) Situation: rigid transition policies, chaotic time management, and unstandardized placement procedures; (2) Self: a role shift from the management core to the functional periphery, creating a mismatch between personal work experience and new job demands; (3) Support: an institutional absence of organizational recognition and financial compensation, countered by the compensatory role of social networks; and (4) Strategies: proactive spatial isolation, reconstruction of workplace power boundaries, and the development of a new professional second curve through job crafting.

**Conclusions:**

The transition of senior head nurses from managerial to nonmanagerial roles is a complex process involving identity reconstruction, loss of managerial authority, and adaptation to new professional expectations. Successful adaptation was facilitated by redefining professional value and actively reshaping work roles. Organizational strategies that support transition planning, recognize prior leadership contributions, and utilize the expertise of former nurse leaders may promote workforce sustainability.

**Implications for Nursing Management:**

This study provides critical insights for nursing workforce management. Healthcare organizations should integrate the transition of senior head nurses into formalized career management frameworks. Furthermore, institutions must implement formal recognition mechanisms for past contributions to enhance organizational belonging. Finally, nursing administrations should establish dual‐track career pathways, including advisory or educational roles. These strategies address perceived overqualification and maximize the continued utilization of senior nursing human capital.

## 1. Introduction

Currently, global healthcare systems are confronting the challenge of an aging nursing workforce, with a substantial cohort of senior head nurses entering the middle‐to‐late stages of their careers [1, 2]. As senior head nurses approach the later stages of their careers, many face transitions from managerial to nonmanagerial roles following organizational restructuring, retirement preparation, or leadership succession [3]. In recent years, in China, hospitals have gradually introduced role adjustment policies for senior nursing managers to support their transition and renewal of nursing management personnel and create opportunities for emerging nurse leaders to develop and advance their careers [4, 5]. Under these policies, once nursing managers reach the age threshold specified by their hospitals, they are required to step down from their managerial positions and transition to nonmanagerial roles [5, 6]. However, these policies vary across hospitals and remain at an exploratory stage. In this study, “senior head nurses” refers to clinical nursing managers who have substantial clinical and managerial experience, have served as head nurses for at least 5 years, and are aged 48 years or older according to the age‐related job adjustment policies of the participating hospitals.

Within this institutional context, reaching the age threshold and stepping down from the head nurse position constitutes a professional transition situated within a specific organizational context. Professional transition refers to the process through which individuals adapt to changes in professional roles, responsibilities, identities, and work environments, involving continuous interaction between personal characteristics, situational factors, support systems, and coping strategies [7]. Given the impact of China’s delayed retirement policy, senior head nurses generally reach retirement age at around 55–58 years [8]. Consequently, most senior head nurses continue working in their original hospitals after stepping down from their head nurse positions and move into different roles, including administrative positions, educational or research management positions, specialized or integrated medical–nursing outpatient clinic positions, and clinical nursing positions within their original departments [9, 10]. The transition was institutionally required, and participants had very limited ability to choose their subsequent positions. It was also accompanied by changes in professional responsibilities, managerial authority, and compensation and employment‐related benefits [11, 12]. Although this transition was institutionally anticipated, its timing and implementation could vary, and the period available for individual preparation could be limited [13]. This transition is far more than a routine handover of duties; rather, it is a complex process that needs rapid psychological adaptation and a comprehensive reconstruction of professional boundaries, role expectations, and interpersonal networks [14, 15]. Particularly under conditions of abrupt reassignment or inadequate organizational support, senior head nurses become highly vulnerable to severe role conflict and maladaptation [16, 17].

Despite its critical implications for workforce sustainability and leadership succession, research on the professional transitions of senior head nurses remains limited. First, existing studies have focused primarily on nurses’ progression into leadership positions [18, 19], while the experiences of those transitioning out of management roles have received little attention. Furthermore, while some studies address general role adaptation, few have explored the psychological evolution and professional identity reconstruction of this downward transition among senior head nurses [20]. In addition, there is a notable lack of in‐depth analysis grounded in systematic professional transition theories [21]. Consequently, understanding the experiences and adaptation processes of senior head nurses during managerial role transition is essential. These insights may guide the development of transition support mechanisms that facilitate role reconstruction, retain valuable leadership expertise, and support effective leadership succession within nursing services [21, 22].

Schlossberg’s 4S transition theory (Situation, Self, Support, and Strategies) posits that an individual’s adaptation to a professional transition is shaped by the dynamic interplay of four dimensions: the specific context of the situation, individual traits and psychological resources (Self), external support systems, and coping strategies [7]. It provides a comprehensive lens for understanding the mechanisms of adaptation during role transitions. Given that the professional transition of senior head nurses entails not only shifts in administrative duties but also profound adjustments in professional identity, organizational dynamics, and psychological states, the 4S model offers a robust and systematic theoretical perspective for analyzing their subjective experiences, support needs, and coping mechanisms. Therefore, Schlossberg’s 4S transition theory was employed to guide the exploration and illumination of the lived experiences of senior head nurses during their professional transitions, providing an in‐depth analysis of their current organizational support systems and the individual coping strategies they adopt.

## 2. Methods

### 2.1. Study Design

This study adopted a descriptive qualitative research design, which aims to provide a rich and contextually grounded account of a phenomenon within a specific sociocultural context [23, 24]. This design is well suited to the study’s aim of exploring how senior head nurses experience and navigate managerial role transitions. The study was conducted and reported in accordance with the Consolidated Criteria for Reporting Qualitative Research (COREQ) guidelines (Supporting File_1) [25].

### 2.2. Setting

This study was conducted in four large tertiary public hospitals located in a provincial capital city in central China, all of which are currently undergoing structural turnover of senior head nurses. Although situated within the same city, which ensured a broadly comparable policy environment, the four hospitals differed in size, departmental composition, and the specific implementation of age‐related transition policies. This combination of contextual similarity and institutional variation provided a foundation for maximum variation sampling, enabling the capture of diverse post‐transition destinations and placement experiences while maintaining analytical coherence across sites.

### 2.3. Sampling and Recruitment

This study utilized purposive and maximum variation sampling strategies to obtain rich information regarding transition experiences. Inclusion criteria were: (1) aged ≥ 48 years, reflecting the range of institutional age thresholds across participating hospitals, some of which had previously applied a threshold of 50 years prior to policy revision; (2) having served as a clinical head nurse for ≥ 5 years, with extensive nursing management experience; (3) having stepped down from the head nurse position and transitioned to a staff nurse or other non‐nursing roles within the past 24 months due to age‐related job adjustment policies; and (4) possessing good communication skills and the ability to clearly articulate their professional transition experiences. Exclusion criteria were: (1) presenting with severe communication barriers or cognitive impairments that would preclude interview completion; and (2) participants who declined to discuss their transition experiences or were unable to provide sufficiently rich data relevant to the study objectives were excluded.

Eligible participants were recruited from the nursing departments of the participating hospitals with support from departmental nurse coordinators, who served a purely logistical role and held no supervisory authority over potential participants. None of the researchers had a pre‐existing professional or supervisory relationship with any participant prior to recruitment. Following approval from the hospital nursing administration, researchers contacted potential participants, briefly explaining the study objectives, interview content, and participation requirements. Those willing to participate were further assessed against the predefined criteria. Semistructured interviews were subsequently arranged according to participants’ availability and preferences. To capture diverse transition experiences, efforts were made to recruit participants with varied post‐transition positions, clinical backgrounds, and years of managerial experience. The sample size was determined based on the principle of data saturation, defined operationally as the point at which three consecutive interviews yielded no new codes or themes.

Of the 25 potentially eligible senior head nurses approached, 2 did not meet the inclusion criteria and 3 declined participation. Two additional individuals participated in pilot interviews to refine the interview guide and were not included in the final analysis. Consequently, 18 participants completed the formal interviews and were included in the final analysis (Figure 1).

**FIGURE 1:**
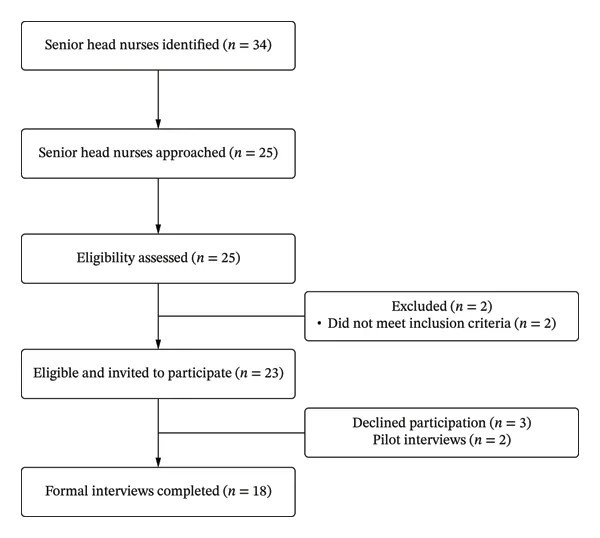
Participant recruitment and inclusion flowchart.

### 2.4. Data Collection

Qualitative data were collected through individual semistructured interviews from March to April 2026. The interview guide was developed based on relevant literature [26–28], research group discussions, and pilot interviews conducted with two participants who were not included in the final sample; the guide was refined following these pilots before data collection commenced.

The semistructured interviews were conducted by two researchers (YG and WC). WC is a nursing professor, and YG is a graduate student in nursing. Both researchers had backgrounds in nursing and experience in qualitative research. Before data collection, they were familiarized with the study objectives, interview guide, and procedures for conducting semistructured interviews. All four participating hospitals were affiliated teaching hospitals of the researchers’ university, and access to the hospitals was obtained through their respective nursing administrations.

Guided by Schlossberg’s 4S transition model [7], the interview questions primarily explored participants’ transition experiences across four domains: (1) Situation: the background and initial reactions upon receiving the transfer notice; (2) Self: individual traits and psychological resources post‐transition; (3) Support: current support from the organization, colleagues, and family; and (4) Strategies: specific ways to cope with challenges in the new position. Open‐ended questions and probing prompts were used to encourage participants to describe their experiences in depth. The full interview guide is provided in Supporting File_2. Interviews were conducted in participants’ offices, private meeting rooms, or other participant‐selected locations to create a comfortable environment and encourage rich descriptions of their transition experiences. All interviews were audio‐recorded and transcribed in Chinese by two researchers (YG and WC) whose first language is Chinese and are fluent in English, with participants’ informed consent. The interviews lasted an average of 45 min. Additionally, the researchers took real‐time field notes on participants’ nonverbal behaviors.

### 2.5. Data Analysis

Data collection and analysis were conducted concurrently, with data analyzed using an inductive thematic analysis approach. Within 24 h of each interview, audio recordings were transcribed verbatim and cross‐checked by a second researcher for accuracy. Field notes documenting participants’ nonverbal behaviors and emotional expressions were incorporated to enrich contextual understanding. Data were managed and coded using NVivo 12.0.

Braun and Clarke’s six‐phase thematic analysis framework was applied [29]. All themes were derived inductively from the data rather than being based on a priori categories. Two researchers (YG and WC) independently read the transcripts repeatedly, conducted open coding, and identified meaningful units related to professional transition experiences. Codes were compared, refined, and grouped into categories through iterative team discussions, from which preliminary themes were derived. Where discrepancies arose between the two coders, a third team member (JC) was consulted and consensus was reached through structured discussion and repeated review of the source transcripts. The transcripts were analyzed in Chinese, and the resulting codes, themes, and interpretations were translated into English for presentation and discussion within the research team. The primary researcher worked with a professional English translator to translate the codes, themes, and interpretations into English. A team member (YL) who was fluent in both Chinese and English reviewed all translated materials to confirm the accuracy and consistency of the English translations with the original Chinese codes and interpretations.

Throughout the analysis, emerging interpretations were continuously compared against the original data, and disconfirming cases were grounded in and accurately reflected participants’ experiences, resulting in the thematic framework presented in this study. Only after the inductive thematic analysis had been completed and the thematic structure had been established was Schlossberg’s 4S transition model introduced as a theoretical lens to interpret the findings, rather than as an a priori coding framework.

### 2.6. Positionality and Rigor

The research team comprised nursing professors (JC, WC, and NW) and graduate students (YG, YL, JL, GG, XY, XD, XL, WG, and WL) with experience in nursing and qualitative research. Several members also had clinical or nursing management experience (JC, GG, NW, and XY), facilitating a deeper understanding of participants’ transition experiences. Throughout data collection and analysis, the research team engaged in reflexive discussions to consider how the researchers’ clinical and nursing management experiences, assumptions, and interpretations might influence interactions with participants and the interpretation of the data. Data collection and analysis occurred concurrently, and two researchers (YG and WC) independently coded the data, with discrepancies resolved through discussion and consultation with a third team member (JC) when necessary. Original participant quotations were retained, and discrepant cases were actively examined to enhance credibility. In addition, the analytical process was documented in detail, and maximum variation sampling was used to strengthen the richness and transferability of the findings.

### 2.7. Ethical Considerations

This study was approved by the Ethics Committee of Xiangya School of Nursing, Central South University (No. E2026114). All participants received written information about the study and provided informed consent before the interviews. Participation was voluntary, and participants could withdraw at any time without consequence. To ensure confidentiality, all data were anonymized, with participants identified by codes (P1 to P18). Audio recordings, transcripts, and related data were stored on encrypted devices accessible only to the research team and will be retained and destroyed in accordance with ethical requirements.

## 3. Results

### 3.1. Characteristics of Participants

A total of 18 senior head nurses who had experienced professional transitions were included in this study. There were all female nurses whose ages ranged from 48 to 55 years. Twelve held a master’s degree, and six held a bachelor’s degree. The participants’ clinical nursing experience ranged from 25 to 37 years, with managerial tenures spanning 10 to 24 years. Prior to their transitions, they managed diverse departments, including general surgery, neurology, the emergency department, the operating room, and the central sterile supply department, thereby ensuring robust sample representativeness.

Post‐transition placements were diverse and could be categorized into three main pathways: (1) administrative, educational, or research management roles; (2) specialized or integrated medical‐nursing outpatient clinics; (3) remaining in their original departments as senior clinical nurses. Detailed characteristics of the participants are presented in Table 1.

**TABLE 1:** Characteristics of participants.

Number	Gender	Age (years)	Educational level	Years in nursing	Years in management	Previous department	Current department	Transition date
P1	Female	54	Bachelor	35	20	General Surgery	Comprehensive Evaluation Center	2025/04
P2	Female	54	Master	34	10	Geriatric Neurology	Graduate Department	2025/06
P3	Female	48	Master	29	19	Central Sterile Supply Department	Infection Control Center	2025/01
P4	Female	51	Master	25	16	Endoscopy Center	Endoscopy Center	2025/01
P5	Female	52	Master	34	10	Emergency Department	Emergency Department	2025/02
P6	Female	48	Master	29	17	Orthopedics	Orthopedic Clinic	2025/01
P7	Female	52	Master	35	24	Orthopedics	Orthopedic Clinic	2024/11
P8	Female	53	Master	35	20	Central Sterile Supply Department	Central Sterile Supply Department	2025/02
P9	Female	54	Bachelor	35	20	Hematology	Hematology Teaching and Research Office	2025/04
P10	Female	54	Bachelor	36	19	Neurology	Neurology	2025/02
P11	Female	54	Bachelor	36	13	Oncology	Oncology	2025/01
P12	Female	53	Bachelor	36	12	Hepatobiliary and Thyroid Surgery	Rehabilitation Management Clinic	2025/01
P13	Female	53	Master	33	22	Geriatrics and Respiratory Medicine	Geriatrics and Respiratory Medicine	2025/01
P14	Female	55	Master	37	16	Integrative Medicine	Integrative Medicine	2025/12
P15	Female	55	Master	32	17	Gastroenterology and Urology	Infection Control Center	2025/01
P16	Female	55	Bachelor	37	20	Spine Surgery	Integrated Medical‐Nursing Clinic	2025/06
P17	Female	54	Master	35	15	Operating Room	Operating Room	2025/02
P18	Female	48	Master	26	11	Breast Surgery	Breast Lymphedema Rehabilitation Clinic	2025/02

Guided by Schlossberg’s 4S transition model, this study analyzed in‐depth interviews with 18 participants, focusing on four dimensions: situation, self, support, and strategies. Through inductive analysis, 4 main themes and 12 subthemes emerged. These themes comprehensively capture the situational experiences, self‐perception, perceived support, and coping strategies of senior head nurses during their professional transitions. Based on these findings, a thematic framework illustrating their professional transition and adaptation was developed (Figure 2). Detailed results are presented below.

**FIGURE 2:**
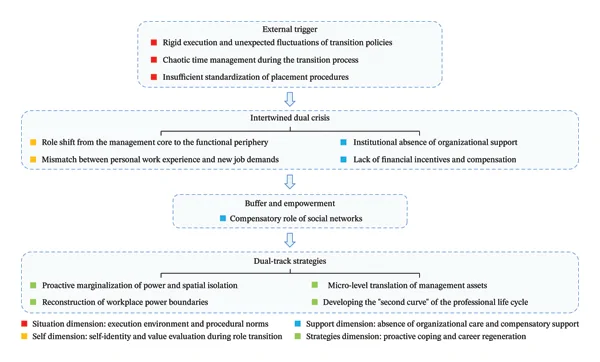
Professional transition and adaptation of senior head nurses: A thematic framework based on Schlossberg’s 4S transition model.

### 3.2. Situation Dimension: Execution Environment and Procedural Norms of the Transition System

Within the Situation dimension, participants predominantly characterized the transition as a mandatory, externally imposed event over which they exercised little to no temporal control. This theme encompasses three subthemes: the rigid execution and unexpected fluctuations of transition policies, chaotic time management during the transition, and insufficient standardization of placement procedures.

#### 3.2.1. Rigid Execution and Unexpected Fluctuations of Transition Policies

Participants experienced the transition as a mandatory process driven unilaterally by hospital policy, with the underlying standards themselves prone to unpredictable change.

This rigidity was most apparent in how transitions were implemented: a “one‐size‐fits‐all” approach based primarily on biological age or administrative tenure, leaving little room for individual circumstances or preferences in the decision‐making process.

“The hospital stipulates that once you reach the age of 50, you can no longer serve as a head nurse.” (P16)

“The main reason for the transition is based on university and hospital requirements. Head nurses need to change positions after serving two terms or more than 8 years.” (P18)

“The reason for the transition is simply a one‐size‐fits‐all policy regarding age. It has nothing to do with my own thoughts and choices.” (P14)

Beyond rigidity, participants highlighted unexpected fluctuations in policy standards. The age threshold for transition was not static; rather, it shifted concurrently with changes in hospital leadership or broader management models. This unpredictability engendered a profound sense of losing control among the senior head nurses.

“The transition was not an expected transfer. Previously, the required transition age was 53. But when new leadership took over that year, it suddenly became 50.” (P15)

#### 3.2.2. Chaotic Time Management During the Transition Process

For participants, the abruptness of decision notifications, compounded by the urgency of work handovers, intensified a pervasive sense of losing control. This subtheme captures two related experiences: an insufficient communication buffer before official notifications, and severely inadequate time for comprehensive handovers.

Hospitals frequently gave nurses little advance notice before announcing transition decisions, leaving no psychological buffer period. In some instances, arrangements were finalized within an exceptionally short time frame, leaving individuals little opportunity to prepare either professionally or psychologically.

“Because we happened to have a hospital inspection in May, they kept rushing me. After I agreed, I had to handle the relevant procedures very hurriedly.” (P1)

“It was very sudden for me. I didn′t proactively say that I absolutely wanted to change positions, but they approached me at that time.” (P2)

Compounding this abruptness was the intense time pressure surrounding work handovers. Hospital administrators or nursing departments often mandated a comprehensive transfer of management duties, team dynamics, and clinical operations within an impractically short window. For individuals transferring across departments or adopting entirely new roles, this truncated handover period fell drastically short of the time required to effectively bridge complex responsibilities.

“One of our senior nurses was asked to finish the handover within three days. Actually, this is too brief. Especially for nurses transferring across departments, this time is insufficient. Many subsequent issues need to be handed over, many situations are not explained, and they are unfamiliar with many things. There are still major drawbacks in this approach.” (P9)

#### 3.2.3. Insufficient Standardization of Placement Procedures

The organization, in participants’ accounts, lacked systematic planning and clear pathways for post‐transition placements. As a result, career destinations depended heavily on informal resources—a key institutional root cause of transition‐related anxiety. This subtheme captures two related experiences: the informal, ad hoc nature of placement pathways, and participants’ clear expectations for systemic improvement.

Hospitals frequently lacked standardized professional transition plans. Individuals were often left to rely on personal networks or to independently secure their next roles.

“Here really weren′t any formal systems or procedures. After stepping down, unless someone happened to know the right people, most of us were pretty much left to figure things out on our own.” (P13)

“There were no arrangements. It was called ′showing your own abilities,′ meaning you just had to find your own way out.” (P11)

Driven by this lack of structure, participants expressed clear expectations for systemic improvement, proposing specific, actionable suggestions that reflected an urgent need for standardized placement procedures.

“I think for this kind of situation, if everyone is transitioning collectively, they should inform us beforehand how each person will be arranged, or what positions are available to choose from. This would give everyone some reassurance. Otherwise, during that period, everyone felt quite anxious and terrified, not knowing where they were going. It is the same for current head nurses facing this now.” (P15)

### 3.3. Self‐Dimension: Self‐Identity and Value Evaluation During Role Transition

Within the self‐dimension, the transition triggered a profound shock to participants’ professional identities. This manifested primarily as a continuous psychological tension between the sense of loss following the deprivation of power and status, and the ongoing reconstruction of their professional value. This theme encompasses two subthemes: the role shift from the management core to the functional periphery, and the mismatch between personal work experience and new job demands.

#### 3.3.1. Role Shift From the Management Core to the Functional Periphery

Following the transition, participants experienced a drastic contraction in their social radius and workplace status—reflected in a sudden loss of decision‐making authority and a significantly diminished professional presence. This subtheme includes two related experiences: the weakening of the core managerial role and the loss of career‐related supporting resources.

The stripping of administrative functions left a psychological gap that generated a deep sense of marginalization among nearly all participants.

“Suddenly, it seems like I am here all alone. The feeling is that you have to go and adapt to the people around you.” (P12)

“I felt that if we stay in the department, we must know that our role has changed. In the past, you were the head of the household in the department, and people would listen to what you said. Now, with the new head nurse taking office, you have to automatically step back into the second line.” (P5)

Beyond formal authority, the loss of career‐related supporting resources and nuanced changes in daily routines further reinforced participants’ awareness of their identity transition.

“In the past, when working on the clinical frontline, it was very convenient to seek medical care for myself. Now that I have withdrawn from clinical practice and no longer wear a nurse′s uniform, I feel it is not as convenient to see a doctor as before.” (P1)

#### 3.3.2. Mismatch Between Personal Work Experience and New Job Demands

Participants generally perceived that the senior management experience they had accumulated over many years was difficult to translate effectively into their new functional positions. This mismatch generated a pronounced sense of efficacy frustration and triggered deep questioning regarding the necessity of the transition. This subtheme encompasses two aspects: the efficacy loss of senior management resources and the sense of loss resulting from the hindered continuation of professional achievements.

Some participants argued that reassigning nursing managers with extensive professional backgrounds to nonclinical functional roles represented an inefficient allocation of human capital—a clear loss of accumulated managerial expertise.

“Actually, for a capable and experienced head nurse like this, going to a logistics department is really a waste of resources. If she does well in her specialty, she can completely use her professional skills to help certain groups of people within her specialty.” (P16)

For other participants, the transition disrupted the continuity of professional achievement: they were forced to relinquish their voice and influence in their original domains and to readapt to unfamiliar specialties and environments. This disruption provoked significant doubts about the rationality of the transition system and, more broadly, about how the nursing profession itself is positioned.

“I did a lot of work in my original professional field and had a certain influence within and outside the province. But now, I have to adapt to a new department and familiarize myself with a new specialty again, including management methods and personnel coordination. I feel it′s hard to utilize my strengths, and I also think there′s no need to change departments.” (P18)

“If you position the nursing profession as a group of workers whose professional skills are not very prominent, then don′t recruit so many master′s and doctoral graduates. The lingering frustration of many people comes exactly from this.” (P14)

### 3.4. Support Dimension: Absence of Organizational Care and Compensatory Support

Within the Support dimension, formal organizational support from the hospital and nursing administration was universally absent. Against this backdrop, social networks played a critical compensatory role, providing essential vocational decision‐making guidance as well as psychological and emotional support. This theme encompasses three subthemes: the institutional absence of organizational support, the lack of financial incentives and compensation, and the compensatory role of social networks.

#### 3.4.1. Institutional Absence of Organizational Support

Participants described a systemic lack of formal organizational support across multiple administrative levels. This subtheme includes two related experiences: the absence of ritual recognition for professional contributions and the lack of mechanisms for transmitting management experience.

The organization failed to formally acknowledge individuals’ professional dedication and cumulative achievements prior to their transition. This omission left participants feeling profoundly neglected by the institution.

“Actually, a sense of ritual is quite necessary. It is to prove that everyone has worked hard for more than half their lives. We still need a sense of ritual for recognition.” (P5)

“During the transition, when the hospital leaders announced the list of new head nurses, they did not say a single word of affirmation for the contributions made by the old head nurses. Nor did they give due recognition in any formal ceremony or official meeting.” (P10)

Beyond the lack of psychological care, the organization also failed to establish effective knowledge transfer protocols for transitioning individuals. As a result, the valuable expertise of senior managers could not be systematically passed on to their successors, producing a structural loss of organizational management capital.

“The nursing department should conduct a comprehensive assessment and let us mentor the new head nurses, rather than just relieving us of our duties without any follow‐up.” (P16)

#### 3.4.2. Lack of Financial Incentives and Compensation

Participants consistently reported a substantial reduction in material benefits following the transition. This subtheme captures two related experiences: the direct decline in financial compensation, and the resulting sense of organizational unfairness.

The shrinkage in performance‐based income after the transition was one of the most immediate changes participants experienced.

“There is no humanistic care; instead, the benefits and allowances have decreased. I feel a strong sense of inequity.” (P17)

“Having stepped down, bonuses and everything else have decreased.” (P11)

This reduction in income was not perceived merely as a material loss. Rather, participants interpreted it as a clear sign that the organization did not value or care for them, which triggered a deep sense of psychological imbalance.

“Because when previous head nurses stepped down in the past, they would receive a certain allowance. But we didn′t get any. We feel it′s unfair.” (P5)

#### 3.4.3. Compensatory Role of Social Networks

Against the backdrop of scarce formal organizational support, participants primarily sought motivation and direction for professional reconstruction through social networks. This subtheme encompasses three distinct aspects: validation from leaders, decision‐making navigation from mentors, and emotional grounding from family.

Validation from leaders took the form of proactive recognition from key decision‐makers, which participants experienced as a rare and vital form of professional respect. This recognition effectively offset the marginalization anxiety they felt during the early phase of the transition.

“The current office director said at the annual meeting that he specifically recruited me into the department. This kind of recognition is extremely important to me.” (P1)

That night, a director called me and said the reason he called so late was that he was afraid I would be snatched away by others. It was indeed quite sudden, because I felt that if I wanted to change positions on my own, it might actually be quite difficult.” (P2)

Mentors also played a navigational role: respected senior colleagues offered objective analyses of the pros and cons of different options, helping participants make career choices better aligned with their personal traits and providing a crucial sense of direction and psychological support.

“I asked my former teacher, and he told me to come to this current position. That gave me confidence. (P1)

“I sought my mentor′s advice. She analyzed the pros and cons of each position with me, and then said that my personality was still suited for staying in clinical practice.” (P4)

Family members provided a different kind of support, helping participants navigate the psychological shift from a demanding managerial role back to a more balanced personal life by validating their years of hard work and offering timely reassurance about the boundaries of their new role.

“Chatting with my sister provided me with great psychological support. She felt that leaving clinical practice was the right choice.” (P1)

### 3.5. Strategies Dimension: Coping and Regeneration

Within the Strategies dimension, participants demonstrated a proactive coping orientation. Through intentional physical spatial detachment and the reconstruction of workplace behavioral boundaries, they achieved a seamless transfer of managerial authority while simultaneously repositioning their personal value to foster professional regeneration. This theme encompasses four subthemes: proactive marginalization of power and spatial isolation, reconstruction of workplace power boundaries, micro‐level translation of management assets, and the development of the “second curve” in the professional life cycle.

#### 3.5.1. Proactive Marginalization of Power and Spatial Isolation

Some participants who remained in their original departments after stepping down from the head nurse position described a symbolic link between the physical office environment and their former managerial identity. Although relinquishing the head nurse position and associated managerial space was institutionally required, their established authority and influence within the department could persist beyond the formal role. These participants therefore described deliberately distancing themselves from the former managerial environment to avoid interfering with the new head nurse’s management and to establish appropriate role boundaries. This subtheme encompasses two aspects: withdrawal from the former physical office environment and proactive distancing from the original managerial social circles. Regarding the withdrawal from the office environment, most participants opted to vacate their original territory promptly. Through complete spatial detachment, they created an intact symbolic power space for the incoming manager, thereby mitigating the transitional awkwardness caused by a lingering presence.

“I moved out of the office very early, leaving the space for the newly appointed head nurse.” (P13)

“I stayed in that office for over 14 years, so I still have feelings for it. But as soon as the new head nurse took office, I immediately took my things away.” (P10)

Participants also distanced themselves from former social circles, consciously minimizing any potential interference with the new manager’s autonomy by increasing physical distance and reducing unnecessary contact.

“After my transition, I went to the old campus to keep some distance from them. Because they are all in the new campus, keeping a distance makes it easier for them to manage. They can manage and reform however they want.” (P8)

“For example, now that I am no longer the head nurse and the department has a new one, I rarely visit the new head nurse′s office.” (P10)

#### 3.5.2. Reconstruction of Workplace Power Boundaries

To maintain organizational structural stability and respect the new manager’s authority, participants consciously redrew their power boundaries during daily interactions. They successfully achieved a role repositioning from formal leader to supportive collaborator. This subtheme encompasses two aspects: the proactive suppression of management intervention impulses and the proactive maintenance of collaborative relationships with successors.

Participants also worked to suppress the impulse to intervene, navigating interactions with their successors in a notably restrained manner and actively avoiding habitual, experience‐based interventions that might hinder the new managers’ independent decision‐making.

“You cannot give orders as you used to when you were the head nurse. Now you can only speak tactfully, or remind them half‐jokingly, telling them how to do it or where the risks are.” (P8)

At the same time, participants demonstrated high levels of professionalism and empathy in maintaining collaborative relationships with their successors, viewing support for the new managers’ smooth adaptation not merely as an organizational courtesy but as a crucial part of their own professional transition.

“We need to put ourselves in others′ shoes. We must consider not only others but also the newly appointed head nurse. They might encounter challenges. We need to think about how to cooperate with them, help them overcome difficulties, and then propose some of our own ideas.” (P5)

“If the new head nurse genuinely recognizes you and proactively seeks advice, as an experienced former head nurse, I will definitely assist them with management without reservation.” (P16)

#### 3.5.3. Micro‐Level Translation of Management Assets

Rather than opting for passive withdrawal, participants actively sought opportunities for value regeneration within their new professional contexts, demonstrating remarkable professional resilience. This subtheme encompasses two aspects: the translation of management experience into micro‐level individual services and the conversion of expert reputation into departmental public relations resources.

Many participants translated their management experience into micro‐level individual services, flexibly adapting prior macro‐coordination skills to optimize individual diagnostic and treatment processes and improve service quality. By substituting macro‐management with precise, patient‐centered care, they reconstructed a sense of professional meaning.

“I will help patients integrate three or four examinations together, saving them extra trips. This is my value now.” (P10)

“When handling patient complaints, I use the experience from when I managed the department, and the results are very good.” (P1)

Others converted their professional reputation into a resource for departmental public relations, naturally assuming the role of informal interdepartmental coordinators by drawing on the extensive cross‐departmental networks and deep professional credibility they had built over many years.

“Having been a head nurse for so many years, it′s relatively easy for me to deal with head nurses and doctors from various departments. They are all willing to have me communicate for them.” (P15)

#### 3.5.4. Developing the “Second Curve” of the Professional Life Cycle

Many participants adopted a forward‐looking and enterprising stance to open up a “second curve” in their career development. Through continuous learning, scientific research innovation, and the deepening of professional expertise, they injected new growth momentum into their professional lives. This subtheme encompasses two aspects: continuous learning and transitional certifications, and scientific research innovation coupled with the deepening of professional expertise.

Some participants pursued continuous learning and transitional certifications, proactively analyzing industry trends and potential future social needs to build human capital through the lateral expansion of their professional skills.

“I actually obtained a caregiver certificate. If this is covered by medical insurance in the future, a large number of nursing assistants will enter clinical practice. Then I can be their trainer. I have the certificate. If society needs it, I can do these things.” (P10)

“Currently, without being a head nurse, I still have many things to do. I have always been doing my First Responder activities. This year, I was even evaluated as a member of the Hunan Province Science Popularization Expert Database. This is also a recognition for me.” (P5)

Others channeled the increased discretionary time following their transition into scientific research and the deepening of professional expertise, focusing on research output and technical skill accumulation. Through this continuous knowledge creation, they sustained a strong sense of professional mission.

“I recently applied for an invention patent and am working on projects that I hadn′t finished before.” (P1)

“Now I am undertaking an apprenticeship in traditional Chinese medicine. I won′t let myself completely detach from professional techniques.” (P14)

## 4. Discussion

Guided by the 4S transition model, this study explored the lived experiences and adaptation processes of senior head nurses transitioning from managerial positions to staff nurse or nonmanagerial roles within Chinese hospitals. The findings suggest that the institutional transition context, compounded by perceived overqualification, significantly diminished participants’ perceived organizational support during the transition. In response, participants adopted two complementary coping strategies to facilitate their adaptation: the proactive relinquishment of power and job crafting. Through these mechanisms, they gradually reconstructed their professional identities and adapted to their new roles. These findings contribute to a deeper theoretical understanding of professional transitions among senior nursing leaders and provide practical insights for developing robust organizational support and tailored professional transition programs.

According to the Situation dimension of the 4S transition model, the initial adaptation difficulties experienced by senior head nurses were closely related to the institutional arrangements governing their exit from managerial positions [10]. In the participating hospitals, age‐related arrangements for leaving the head nurse position varied in terms of the applicable age threshold, timing of policy issuance, and implementation procedures. These arrangements therefore represented a hospital‐specific institutional context rather than a uniform policy. In some cases, the transition criteria changed with changes in hospital leadership or management models, while participants in some instances received transition notifications without sufficient time for adjustment and were required to complete the handover of managerial responsibilities within a short period [10, 26]. Organizational responses focused primarily on the administrative handover of managerial responsibilities and subsequent placement [13, 30], while formal support for psychological adjustment, professional preparation, knowledge transfer, recognition of professional contributions, and financial transition remained limited [10, 31]. Although participants generally understood the policy as an institutional arrangement intended to facilitate leadership succession, they perceived its implementation as relatively inflexible and had limited autonomy in determining their subsequent role placement [32]. Meanwhile, unpredictable changes in transition criteria, limited advance notice, and the lack of standardized placement procedures limited participants’ opportunities to make proactive preparations for the transition [33].

Beyond these situational constraints, participants also faced challenges related to the self‐dimension of transition, particularly changes in professional identity and the perceived value of their accumulated expertise. Unlike studies of upward promotion, which often highlight role overload and competence‐related anxiety among newly appointed managers [34, 35], participants in the present study primarily experienced perceived overqualification [36]. They felt that their accumulated knowledge, skills, and managerial experience were underutilized in their new positions. This finding suggests that adaptation challenges following managerial role exit arise not from insufficient competence but from a mismatch between individuals’ qualifications and reduced job demands. Consistent with previous research, perceived overqualification was associated with diminished job satisfaction, weakened professional identity, and increased psychological strain [37–39], helping to explain the disruption to participants’ self‐efficacy and role identity during the early transition period. However, existing evidence also indicates that when organizations provide opportunities to utilize employees’ surplus capabilities, perceived overqualification can become a resource that promotes positive adaptation and work engagement [6, 40].

Support represents a key external resource within the 4S transition model. This study found that the abrupt loss of performance‐related benefits and the absence of formal recognition during managerial role exit significantly weakened participants’ perceived organizational support. Perceived organizational support reflects employees’ global beliefs concerning the extent to which the organization values their contributions and cares about their well‐being [41]. Participants’ expectations regarding honorary recognition and a smoother compensation transition reflected not only material concerns but also a profound desire for organizational acknowledgment, professional value affirmation, and identity continuity. Consistent with previous research, perceived organizational support serves as a vital resource that buffers work‐related stress and facilitates adaptation during professional transitions [42]. When such support is systematically lacking, individuals may experience deep feelings of neglect and diminished professional identity, thereby hindering the transition process [43]. In response to limited formal support, many participants actively sought resources from informal support networks. Encouragement from new department leaders, guidance from senior mentors, and emotional support from family members emerged as crucial sources of compensatory assistance. These forms of support provided both emotional reassurance and professional validation, empowering participants to maintain a positive sense of self and adapt to their new roles [44]. The findings suggest that formal organizational support and informal social support operate as complementary resources that jointly facilitate successful transitions and psychological adaptation among senior head nurses.

In the face of the situational pressures and limited support resources described above, participants did not resort to passive withdrawal. Instead, they demonstrated an active adaptation process characterized by the deliberate relinquishment of former roles and the proactive reconstruction of new professional identities. Many participants intentionally vacated their former physical workspaces, reduced contact with their previous departments, and abstained from intervening in managerial affairs post‐transition. Unlike previous studies suggesting that departing managers may resist relinquishing authority due to insecurity or power entrenchment [30], participants in this study viewed disengagement from their former roles as a deliberate and responsible strategy. Consistent with the findings of Pilat et al., they consciously ceded space for their successors out of respect for departmental staff and a commitment to organizational stability [45]. By strategically withdrawing from their previous spheres of influence, they minimized potential conflicts arising from overlapping authority and facilitated a smoother leadership transition [46].

Beyond the strategic relinquishment of their former managerial roles, participants actively engaged in specialized nursing practice, patient communication, nursing education, and research activities. Consistent with previous studies, they employed job crafting to proactively reshape their task boundaries and cognitive framing, thereby enhancing their sense of meaning and control [6, 47, 48]. Drawing on accumulated clinical expertise, managerial acumen, and psychological capital, participants expanded into novel areas of practice and reconfigured their strengths to meet evolving job demands. These findings suggest that the transition of senior head nurses is not merely an identity replacement, but a profound process of professional role reconstruction [49]. By translating their managerial experience, mentoring capabilities, and professional knowledge into new work contexts, participants continued to generate value beyond formal leadership boundaries and successfully regained their sense of professional worth.

Drawing upon these findings, several practical implications for nursing workforce management emerge. For senior head nurses subject to age‐related transition policies, the findings highlight the importance of anticipating role transition, identifying opportunities to transfer accumulated expertise, and preparing for professional roles beyond formal managerial positions. First, to mitigate the loss of environmental control triggered by institutional transition arrangements, healthcare organizations should integrate the transition of senior head nurses into a formalized career management framework. Establishing standardized transition timelines, transitional buffer periods, and structured handover procedures will significantly enhance the predictability of role shifts and facilitate psychological adaptation. Second, to counteract the decline in perceived organizational support during the transition, institutions must establish formal recognition mechanisms for professional contributions. Acknowledging cumulative achievements through equitable compensation adjustments, formal transition recognition, and other honors will substantially reinforce individuals’ sense of professional worth and organizational belonging. Third, to address perceived overqualification and catalyze professional role reconstruction, nursing administrations should establish dual‐track career pathways. Empowering transitioning head nurses to sustain their impact through specialized clinical, educational, advisory, or quality‐improvement roles will enable them to translate their accumulated managerial expertise into professional influence, thereby maximizing the continued utilization of senior nursing human capital. For nurses in healthcare systems without age‐based restrictions on managerial tenure, the findings nevertheless highlight the broader importance of organizational support, preparation for late‐career role transitions, and opportunities to apply accumulated expertise when moving out of formal leadership positions.

The primary contribution of this study lies in its exploration of the transition experiences and support needs of senior head nurses following institutionally required exit from managerial roles due to age‐related policies in the Chinese healthcare context. It also provides practical strategies for supporting such role transitions. Although the findings are rooted in the Chinese context, they may offer valuable insights for other healthcare systems seeking to address late‐career role transitions and support experienced nursing personnel in continuing to contribute professionally beyond formal managerial positions.

Several limitations should be acknowledged. First, the cross‐sectional qualitative design did not permit examination of how adaptation experiences evolve across different stages of transition. Second, participants were recruited exclusively from four tertiary hospitals in central China and were all female, which may limit the transferability of the findings to other organizational contexts and male head nurses. Third, the findings should be interpreted within the context of the age‐related institutional policies and employment arrangements of the participating hospitals. They may have limited transferability to contexts in which senior head nurses leave their managerial positions because of retirement, lateral reassignment, or a personal decision to return to clinical practice. Future research could employ longitudinal and mixed‐methods designs to explore the dynamic process of transition adaptation and evaluate the effectiveness of organizational support strategies in facilitating successful professional transitions among senior nursing leaders.

## 5. Conclusion

This study advances understanding of the transition from managerial to nonmanagerial roles among senior head nurses. Guided by Schlossberg’s 4S transition model, the findings demonstrate that adaptation is shaped by the dynamic interaction of transition circumstances, personal perceptions, available support, and coping strategies. Participants experienced challenges related to role loss, perceived underutilization of expertise, and limited formal organizational support, yet actively reconstructed their professional identities through redefining professional value and reshaping work roles. These findings suggest that managerial role transition is not merely an organizational reassignment but a complex process of identity reconstruction and professional adaptation. Healthcare organizations should implement structured transition support, including transition planning, formal recognition of prior leadership contributions, and alternative career pathways that retain the expertise of experienced nurse leaders and support workforce sustainability.

## Funding

This study was funded by (1) Hunan Provincial Key Laboratory Project (Grant No. 2017TP1004), (2) National Natural Science Foundation of China Young Scientists Fund (Category C, Grant No. 72504290), and (3) Hunan Provincial Natural Science Foundation‐Young Scientists Fund (Category C, Grant No. 2025JJ60787).

## Disclosure

The funders had no role in the conceptualization, design, data collection, analysis, decision to publish, or preparation of the manuscript.

## Conflicts of Interest

The authors declare no conflicts of interest.

## Supporting Information

Additional supporting information can be found online in the Supporting Information section.

## Supporting information


**Supporting Information 1** Supporting File_1: The completed Consolidated Criteria for Reporting Qualitative Research (COREQ) checklist, documenting the reporting of this study in accordance with the COREQ guidelines.

**Supporting Information 2** Supporting File_2: The full semistructured interview guide used to explore senior head nurses’ experiences of transitioning from managerial to nonmanagerial roles.

## Data Availability

The data are available from the corresponding author upon request.
