# Supplementary material for: “Value Beyond Management”: Professional Transition Among Senior Head Nurses in China: A Descriptive Qualitative Study Based on Schlossberg’s 4S Model
Source: J Nurs Manag. 2026 Oct 23;2026:1907433. doi: 10.1155/jonm/1907433 (PMC13692706; doi:10.1155/jonm/1907433)
Supplement: Supplementary file 1 [file JONM-2026-1907433-s001.docx]

**Interview Guide**

**Situation**

1. Could you tell us about the main reason for your job transition? Was it something you proposed yourself, or was it arranged by the hospital/department?
2. Was this transition a planned move that you had anticipated, or did it come as a surprise?
3. Does the hospital have a clear, formal system and process for managerial role transitions? What does this process typically involve?
4. In terms of your destination or the decision-making process surrounding the transition, how much autonomy or choice did you feel you had?

**Self**

5. When you received the transfer notification (or made the decision to transition yourself), how did you feel at that time?
6. Facing this role adjustment, how did you feel about your own ability to adapt to and perform competently in the new position? Did you experience any lack of confidence or other concerns?
7. Do you feel that the work following your transition brought stress or difficulty adapting? How did you typically regulate or cope with this?
8. What personal qualities, skills, or past experiences do you feel helped you adapt to the new position? Conversely, in which areas did you find it most challenging, or feel there was room for improvement?

**Support**

9. During the transition process, did you receive relevant support from your family, colleagues, the nursing department, or the hospital?
10. Did you encounter any particular difficulties or obstacles during the transition process?

**Strategies**

11. Faced with the new environment and pressures brought about by the transition, what measures did you take to help yourself adapt?
12. Did you experience emotional fluctuations during the transition? How did you manage your emotions—for example, what did you do to stay positive or relaxed?
13. Do you feel your current job allows you to realize your sense of self-worth? In this process, how did you set or adjust new career goals for yourself?
14. For other head nurses who may go through a similar job transition, do you have any advice or experiences to share? For instance, what should they pay particular attention to before or after the transition?
