# Supplementary material for: “Value Beyond Management”: Professional Transition Among Senior Head Nurses in China: A Descriptive Qualitative Study Based on Schlossberg’s 4S Model
Source: J Nurs Manag. 2026 Oct 23;2026:1907433. doi: 10.1155/jonm/1907433 (PMC13692706; doi:10.1155/jonm/1907433)
Supplement: Supplementary file 2 [file JONM-2026-1907433-s002.docx]

**Supplementary File_1: COREQ checklist.**

| **No. Item** | **Guide questions/description** | **Reported** |
| --- | --- | --- |
| **Domain 1: Research team and reﬂexivity** |  |  |
| *Personal Characteristics* |  |  |
| 1. Interviewer/facilitator | Which author/s conducted the interview or focus group? | Interviews were conducted by the first author. |
| 2. Credentials | What were the researcher’s credentials? E.g., PhD, MD | The research team included members with Master's and PhD degrees. |
| 3. Occupation | What was their occupation at the time of the study? | The team consisted of nursing students and nursing professors. |
| 4. Gender | Was the researcher male or female? | Female and Male. |
| 5. Experience and training | What experience or training did the researcher have? | The researcher received systematic training in qualitative research methodology and interview techniques prior to the study. |
| *Relationship with participants* |  |  |
| 6. Relationship established | Was a relationship established prior to study commencement? | We didn’t establish a relationship with participants prior to study commencement. |
| 7. Participant knowledge of the interviewer | What did the participants know about the researcher? e.g. personal goals, reasons for doing the research | Informed Consent Statement. |
| 8. Interviewer characteristics | What characteristics were reported about the inter viewer/facilitator? e.g. Bias, assumptions, reasons and interests in the research topic | The researcher maintained reflexivity and deliberately bracketed personal assumptions regarding managerial transitions to minimize bias during data collection and analysis. |
| **Domain 2: study design** |  |  |
| *Theoretical framework* |  |  |
| 9. Methodological orientation and Theory | What methodological orientation was stated to underpin the study? e.g. grounded theory, discourse analysis, ethnography, phenomenology, content analysis | A descriptive qualitative study design, guided by the Schlossberg's 4S transition model. |
| *Participant selection* |  |  |
| 10. Sampling | How were participants selected? e.g. purposive, convenience, consecutive, snowball | Purposive sampling. |
| 11. Method of approach | How were participants approached? e.g. face-to-face, telephone, mail, email | Face-to-face and via telephone/WeChat initially to establish contact and schedule. |
| 12. Sample size | How many participants were in the study? | 18 participants. |
| 13. Non-participation | How many people refused to participate or dropped out? Reasons? | None. All invited participants agreed to participate and completed the study. |
| *Setting* |  |  |
| 14. Setting of data collection | Where was the data collected? e.g. home, clinic, workplace | Face-to-face interviews were conducted in private, quiet meeting rooms or offices within the participants' respective hospitals. |
| 15. Presence of non-participants | Was anyone else present besides the participants and researchers? | No. |
| 16. Description of sample | What are the important characteristics of the sample? e.g. demographic data, date | Female senior head nurses from four tertiary Grade A hospitals in central China. Of the 18 participants, 12 (66.7%) held a master's degree and 6 (33.3%) held a bachelor's degree. |
| *Data collection* |  |  |
| 17. Interview guide | Were questions, prompts, guides provided by the authors? Was it pilot tested? | A semi-structured interview guide was developed by the research team and pilot-tested with two head nurses prior to the formal study. |
| 18. Repeat interviews | Were repeat interviews carried out? If yes, how many? | No. |
| 19. Audio/visual recording | Did the research use audio or visual recording to collect the data? | Audio Recording. |
| 20. Field notes | Were ﬁeld notes made during and/or after the inter view or focus group? | Yes. |
| 21. Duration | What was the duration of the inter views or focus group? | 30 minutes to 60 minutes. |
| 22. Data saturation | Was data saturation discussed? | Data saturation was discussed and deemed to have been achieved when no new themes emerged from the last three consecutive interviews. |
| 23. Transcripts returned | Were transcripts returned to participants for comment and/or correction? | Yes, transcripts were returned to participants for member checking to ensure accuracy and resonance with their experiences. |
| **Domain 3: analysis and ﬁndings** |  |  |
| *Data analysis* |  |  |
| 24. Number of data coders | How many data coders coded the data? | Two researchers independently coded the data. Discrepancies were resolved through discussion with a third senior researcher. |
| 25. Description of the coding tree | Did authors provide a description of the coding tree? | A coding tree was developed iteratively and refined through team consensus, mapping onto the 4S model dimensions. |
| 26. Derivation of themes | Were themes identiﬁed in advance or derived from the data? | Themes were inductively derived from the data and subsequently mapped onto the deductive framework of the 4S model. |
| 27. Software | What software, if applicable, was used to manage the data? | NVivo 12 software was used to manage and organize the qualitative data. |
| 28. Participant checking | Did participants provide feedback on the ﬁndings? | Participants provided feedback on the preliminary findings, confirming that the themes accurately reflected their experiences. |
| *Reporting* |  |  |
| 29. Quotations presented | Were participant quotations presented to illustrate the themes/ﬁndings? Was each quotation identiﬁed? e.g. participant number | Yes, representative quotations were provided and identified by participant numbers (e.g., P1, P2). |
| 30. Data and ﬁndings consistent | Was there consistency between the data presented and the ﬁndings? | Yes, there is clear consistency between the presented quotes and the synthesized findings. |
| 31. Clarity of major themes | Were major themes clearly presented in the ﬁndings? | Four major themes were clearly identified and presented. |
| 32. Clarity of minor themes | Is there a description of diverse cases or discussion of minor themes? | Twelve sub-themes were explicitly described to capture the diversity of the participants' experiences. |
